# Influence of Climate Variability and Reservoir Operation on Streamflow in the Yangtze River

**DOI:** 10.1038/s41598-019-41583-6

**Published:** 2019-03-25

**Authors:** Yuanfang Chai, Yitian Li, Yunping Yang, Boyuan Zhu, Sixuan Li, Can Xu, Congcong Liu

**Affiliations:** 1 State Key Laboratory of Water Resources and Hydropower Engineering Science, Wuhan University, Wuhan, 430072 China; 2 Key Laboratory of Engineering Sediment, Tianjin Research Institute for Water Transport Engineering, Ministry of Transport, Tianjin, 300456 China; 3 School of Hydraulic Engineering, Changsha University of Science & Technology, Changsha, 410004 China

## Abstract

Understanding the effects of climate variability and reservoir operation on runoff is important for shipping, irrigation and water supply services, especially during extreme drought years. After the operation of the Three Gorges Dam (TGD) began, the discharge processes in the mid-lower reaches of the Yangtze River were completely different from those during the pre-dam period. The measured hydrological data and the Mike 11-HD model were used to estimate the contributions of the TGD operation and climate variability to the variation in discharge during extreme drought years. The results are as follows: under the effects of the TGD operation and extreme drought, the special phenomenon of a “positive discharge anomaly in drought season and negative discharge anomaly in flood season” occurred compared with the conditions in the pre-dam period. During the flood season, the contributions of climate variation (TGD operation) to the changes in streamflow from Yichang station to Datong station were 86.6% (13.4%) and 80.7% (19.7%) in 2006 and 64.8% (35.2%) and 71.3% (28.7%) in 2011. During the dry season, the values were 81.2% (18.8%) and 93.9% (6.1%) in 2006 and 59.9% (40.1%) and 72.9% (27.1%) in 2011. Clearly, climate variation was the main reason for the variation in seasonal runoff. Furthermore, even in the 156 m and 175 m impoundments, climate variation was the dominant factor.

## Introduction

Analysing the causes of streamflow variation is of great significance to environmental protection, economic development and social stability. A sudden decrease of streamflow can have a large negative impact on the distribution of aquatic plants and animals as well as shipping and irrigation; furthermore, the significant increase of runoff can result in flood disasters that seriously threaten lives and property. Under the background of global warming, extreme drought events have been occurring much more frequently than before^1,2^, and these events have received considerable attention^3,4^. These events have caused the death of millions of people and livestock^5^. Furthermore, runoff variation is more sensitive to climate variability and human activities during extreme drought years.

The variation characteristics of discharge become more complex under the effects of both climate variability and reservoir operation. For instance, glaciers represent important water resources and significantly contribute to streamflow^6^. It is estimated that glacier sources contribute to at least 10% of river discharge on a seasonal basis^7^. With warmer temperatures around the world, glaciers are predicted to retreat earlier, which can significantly affect runoff seasonality, especially during extreme drought years^8,9^. As a result, the spring discharge is expected to increase, and the summer discharge is predicted to decrease. Apart from the significant effects of climate variability on river discharge, reservoir operation also plays a crucial role in the variation of inner-annual runoff distribution^10^. The reservoirs that implement annual flow regulation will increase the amount of discharge downstream from the dam to meet the needs of irrigation, ecological protection, shipping and combating drought in the dry season; in contrast, the reservoirs can reduce the discharge to prevent downstream floods in the flood season.

**Figure 1:**
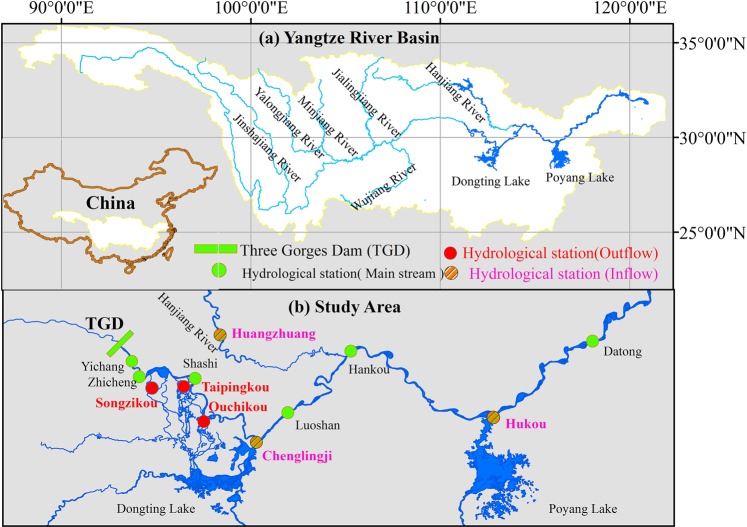
(**a**) Simplified map of the geographical location; (**b**) the study area with the geographical locations of the hydrological stations mentioned in the study.

In 1978, 1986, 2006 and 2011, the Yangtze River Basin suffered extreme drought, and because of this, the annual average discharge at the Yichang, Hankou and Datong stations decreased significantly (*for more details, see* Fig. 1 *in SI*). Thus, the discharge process was changed in the mid-lower reaches of the Yangtze River (MLRYR) due to extreme drought events. Furthermore, the Three Gorges Dam (TGD), the largest reservoir in the world^11^, has changed the discharge timing in the MLRYR since 2003^12–14^. More specifically, in the flood season, the 156 m (the water level behind the TGD increased from 135 m to 156 m) and 175 m (the water level behind the TGD increased from 156 m to 175 m) impoundments were completed in 2006 and 2011, respectively, which further reduced discharge and altered discharge timing. Therefore, under the effects of both the extreme drought events and the operation of the TGD, the variation characteristics of discharge in the extreme drought years of 2006 and 2011 (i.e., post-TGD) were completely different from those in the extreme drought years of 1978 and 1986 (i.e., pre-TGD).

In addition to the special variation characteristics of discharge in 2006 and 2011, the extreme drought climate in these two years caused considerable negative effects. Taking the year 2011 as an example, the direct economic losses in Anhui Province were estimated at 0.58 billion dollars, and more than 266 small-scale reservoirs dried up (http://www.wanfangdata.com.cn/details/detail.do?_type=conference&id=7653371). Reservoir operations can have a significant effect on the drought degree downstream of the dam. For instance, the construction of upstream dams aggravated the downstream drought in the Diyala River basin^15^. The TGD’s initial operation from 2003 to 2011 also aggravated the hydrological droughts at the downstream Yichang station^16^. Thus, due to the large social effects and the relevant research results, many people have started to suspect that the frequent drought events since the TGD began operating, especially the extreme drought events in 2006 and 2011, might be attributed to the operation of the TGD^17^. A public question about “whether the TGD operation should be responsible for the extreme drought in 2006 and 2011”needs to be explained in detail.

In the Yangtze River Basin, most studies have concentrated on assessing the influence of climate variation and human activities on the changes in the annual runoff^11,18,19^ or sediment load^20–25^ before and after the TGD began operating, while few studies have been conducted on the effects of extreme drought climate and reservoir operation on the changes in seasonal runoff distribution^26,27^ between dry and flood seasons; furthermore, little research has been done to quantify the contributions of these two factors. After the TGD began operating, the seasonal runoff distribution was significantly changed compared to that in the pre-TGD period. By ignoring the effects of climate change, the existing research results show that the TGD operation is the reason for the variation in seasonal runoff^28^. The question of whether the operation of the TGD is responsible for the changes in the seasonal runoff distribution is still unknown when the influences of climate changes are considered, especially during the extreme drought years.

Based on the unusual variation characteristics of discharge in 2006 and 2011 and the public question stated above, the main aims of this study are as follows: 1) to analyse the variation characteristics of discharge in the MLRYR and its causes by comparing values with the extreme years of 1978 and 1986; 2) to analyse whether the operation of the TGD is responsible for the extreme droughts in the MLRYR in 2006 and 2011; and 3) to quantify the contributions of the operation of the TGD and climate variability to the variation in discharge using the Mike 11-HD model. The calculated contributions will be practically significant to optimize reservoir operation rules and water resource distribution and management.

## Study Area

The Yangtze River originates from the Qinghai-Tibetan Plateau and flows for approximately 6397 km into the East China Sea (Fig. 1a), and its total drainage area is approximately 1.8 × 10^6^ km^2^. In addition to the largest reservoir in the world, namely, the TGD, more than 50,000 reservoirs have been built since 1950, and the total capacity is 159 × 10^8^ m^3^. The river is separated by the Yichang and Hukou hydrological stations into the upper reach, the middle reach and the lower reach, primarily on the basis of geology and climate and secondarily on the basis of the resulting geomorphology of the river. The main stream above the Yichang station, where the TGD is situation, is the upper reach, with a length of 4504 km. The middle reach is from Yichang station to Hukou station and has a length of 955 km, and the lower reach is below Hukou station and has a length of 938 km. There are two lakes and a main tributary in the MLRYR: Dongting Lake, Hanjiang River and Poyang Lake. The runoff of the Yangtze River flows into Dongting Lake through three mouths, namely, Songzikou, Taipingkou, and Ouchikou (the red circles in Fig. 1b). The supplements of runoff from Dongting Lake, Hanjiang River, and Poyang Lake into the Yangtze River are gauged at the Chenglingji, Huangzhuang and Hukou stations, respectively (the brown circles in Fig. 1b).

## Results

**Figure 2:**
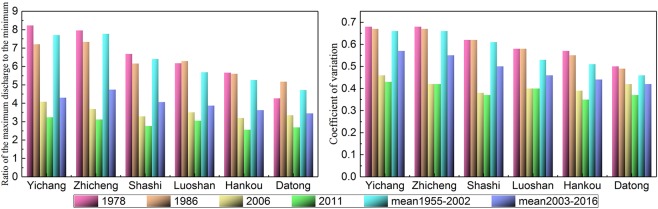
Statistical parameters of the ratio of the maximum discharge to the minimum discharge and thecoefficient of variation at the six hydrological stations.

**Figure 3:**
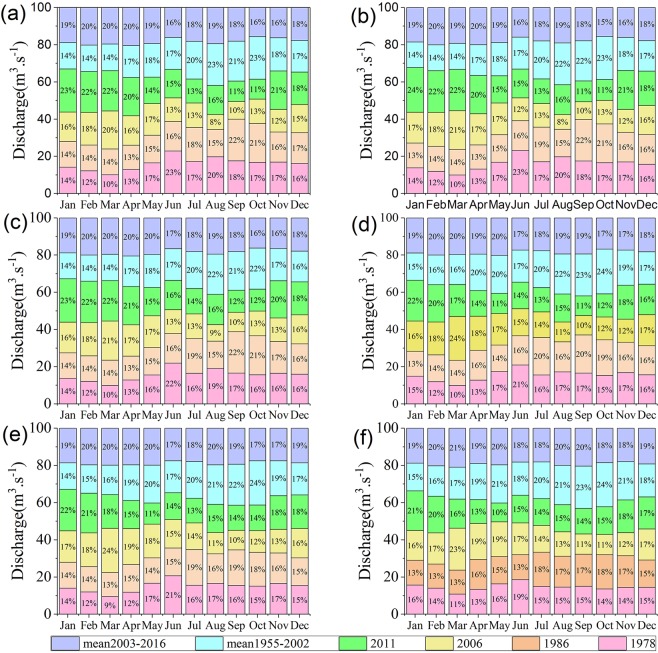
The 100% stacked columns of monthly average discharge at Yichang station (**a**), Zhicheng station (**b**), Shashi station (**c**), Luoshan station (**d**), Hankou station (**e**) and Datong station (**f**) during typical years. Note: each stacked column is composed of six stacked elements, and the cumulative proportion of the six stacked elements is always equal to 100%. Each stacked element is the ratio of the monthly average discharge during a typical year to the sum of the monthly average discharges during six typical years (mean 2003–2016, mean 1955–2002, 2011, 2006, 1986 and 1978). The larger proportions during the dry seasons in 2006 and 2011 represent “positive discharge anomalies in the drought season”, and the small values during the flood seasons in 2006 and 2011 indicate “negative discharge anomalies in the flood season”.

The ratio of the maximum discharge to the minimum discharge and the coefficient of variation (Fig. 2) represents the degree of spread of the inner-annual discharge, and those in the extreme drought years of 1978 and 1986 are larger than the overall average from 1955 to 2002, which means that the difference in the discharge between seasons in the MLRYR has increased due to extreme drought. In contrast, the two statistical parameters were significantly smaller from 2003 to 2016 than from 1955 to 2002, which can lead to the conclusion that the amplitude of variation in the discharge was obviously reduced due to the TGD. In 2006 and 2011, under the combined effects of extreme drought and the TGD, the two statistical parameters, in particular, were the smallest compared with other typical years, implying that the discharge processes in 2006 and 2011 were the smoothest. Therefore, this obvious and peculiar variation in discharge characteristics in the MLRYR in 2006 and 2011 is described here as a “negative discharge anomaly in the flood season, and positive discharge anomaly in the drought season”(NDFS-PDDS). As shown in Fig. 3, the percentage values of the monthly average discharge at six hydrological stations in 2006 and 2011 appear as a saddle shape, with “small values in the flood season and large values in the dry season”, and these results correspond to the unusual phenomenon of “NDFS-PDDS”. In contrast, the percentage values in 1978, 1986, 1955–2002 and 2003–2016 were “large in the flood season and small in the dry season” (Fig. 3).

### Discharge in flood season (May to October)

As shown in Fig. 3, the percentage values of the monthly average discharge at six stations in the flood seasons in 2003–2016 illustrated a significant fall compared with those in 1955–2002, which means that the operation of the TGD reduced the streamflow during the flood season. Under the influence of extreme drought, the percentage values in 1978 and 1986 showed a slight downward trend compared with those in1955–2002. Under the effects of both the operation of the TGD and the extreme drought, the percentage values were the smallest in 2006 and 2011compared to those in 1978, 1986, 1955–2002 and 2003–2016, which indicates that the streamflow during the flood seasons in 2006 and 2011 significantly decreased compared with the other typical years. Based on the observed data, the average discharge during the flood season at the Yichang, Zhicheng, Shashi and Hankou stations in 2006 decreased to the lowest point in history (1955–2016), and at Luoshan and Datong stations in 2011 both dropped to their lowest points in history. Compared with 1978 and 1986, the maximum decreasing value of the average discharge in 2006 and 2011 was up to 7580 m^3^∙s^−1^ (Table 1). Clearly, this obvious phenomenon of NDFS occurred in 2006 and 2011.

**Table 1:** The average discharge during flood and dry seasons (m^3^∙s^−1^).

	1978		1986		2006		2011		1955–2002		2003–2015	
	Flood season	Dry season	Flood season	Dry season	Flood season	Dry season	Flood season	Dry season	Flood season	Dry season	Flood season	Dry season
Yichang	19540	5098	18799	5301	12387	5611	13996	7474	21529	5816	18752	6658
Zhicheng	19994	5283	19316	5448	12414	6097	14659	8002	22099	5946	18808	6911
Shashi	16774	5196	17070	5555	11566	6106	13374	7798	18937	5878	17076	6986
Luoshan	24986	8382	25713	9146	18875	10505	18759	10687	29860	10599	26615	11438
Hankou	26787	9332	27010	10225	21469	12309	21827	12967	32644	11968	29549	13225
Datong	30255	12496	31709	13414	27801	15743	26844	15385	39939	16440	36780	17482

### Discharge in the dry season (December to April)

In the dry season, the percentage values at six stations in 2006 and 2011did not change substantially even under the effects of extreme drought, which contrasted with the data of 2003–2016; however, the values presented a notable upward trend compared with those of 1978 and 1986 (Fig. 3). Based on the observed data, the average discharge values during the dry seasons in 2006 and 2011 were approximately the same as the values of 2003–2016, and the difference in the values was between −2097 m^3^∙s^−1^ and 1098 m^3^∙s^−1^ (Table 1). In contrast, the average discharges in 1978 and 1986 showed a remarkable decline in comparison with the average statistic of 1955–2002, with the maximum reduction being as much as −3944 m^3^∙s^−1^. Furthermore, compared with 1978 and 1986, the maximum increasing value of the average discharge in 2006 and 2011 was as high as 3635 m^3^∙s^−1^. Therefore, the PDDS was a distinct variation pattern for discharge in the MLRYR in 2006 and 2011.

Based on the CMIP5 models, many studies have predicted that the precipitation in the Yangtze River in summer (belonging to flood season) will decrease and that in spring (belonging to dry season) will increase during the early 21st century^29,30^. In the future, the potential evapotranspiration in summer will increase significantly, with some models projecting increases as great as 25 mm^31^. This variation trend of precipitation and evapotranspiration will further increase the runoff in the dry season and decrease the runoff in the flood season; thus, the unusual phenomenon might be increasingly frequent.

### Estimating the contributions of the operation of the TGD and climate variability

Due to the combined impacts of the TGD and extreme drought, the peculiar phenomenon of “NDFS-PDDS” occurred in 2006 and 2011 instead of in 1978 and 1986. Thus, Yichang and Datong stations were selected to compare the values in 2006 and 2011 with that in 1978 to calculate the contributions of the TGD operation and climate variability to this special and interesting phenomenon. The discharge of the Yichang and Datong stations is most affected by the operation of the TGD and by climate variability, respectively. The annual average discharges of 1978, 2006 and 2011 at Datong station were the closest to each other, which means that the calculations occurred under the same discharge conditions. Clearly, the selected hydrologic stations and years are representative.

### During the flood season

**Figure 4:**
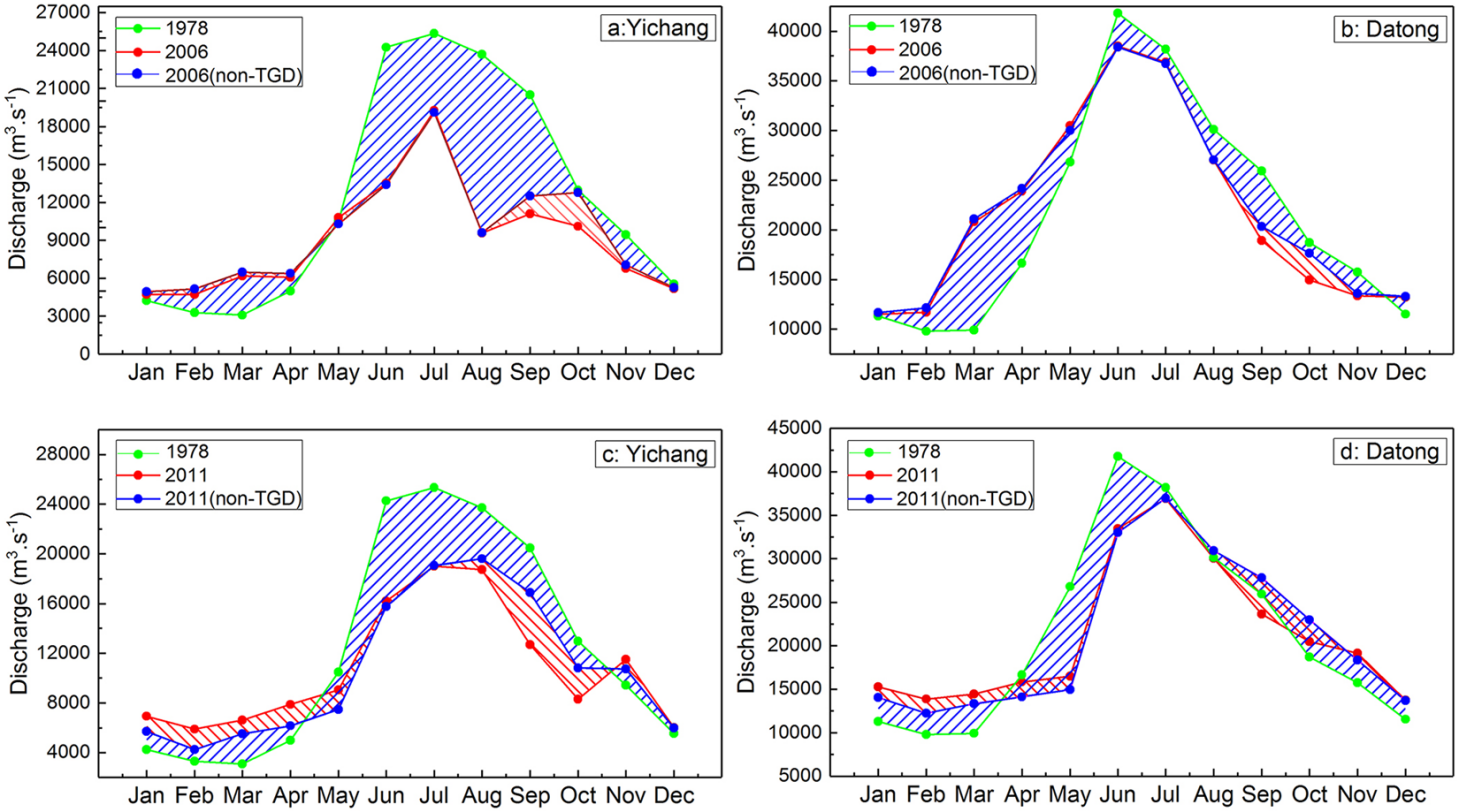
The reconstructed natural monthly average discharge and the actual monthly average discharge at Yichang and Datong stations. Note: the monthly average discharge at Yichang and Datong stations in 2006 and 2011 without the TGD can be reconstructed using the Mike 11-HD model. The green line represents the actual monthly average discharge in 1978; the red line denotes the actual monthly average discharge in 2006 or 2011; and the blue line represents the reconstructed natural monthly average discharge in 2006 or 2011.

As shown in Fig. 4, the reconstructed natural discharge in the flood season did not show an obvious increase compared with the value before restoration. Furthermore, the reconstructed natural discharge had an obvious phenomenon of “NDFS”. The red area between the blue line and the red line shows that the variation in discharge was caused by the operation of the TGD, and the blue area between the blue line and the green line means that the changes in discharge were induced by climate variability. Based on the size of these two areas during the flood season, we can conclude that the contributions of the flood peak reductions at Yichang and Datong stations in 2006 were 13.4% and 19.3%, respectively and those of climate variability were 86.6% and 80.7%, respectively. In 2011, the flood peak reduction accounted for 35.2% and 28.7% of the variation in the discharge at the Yichang and Datong stations, respectively, and the contributions of climate variability were 64.8% and 71.3%, respectively. Thus, the main reason for NDFS was climate variability rather than flood peak reduction.

### During the dry season

As shown in Fig. 4a,b, the reconstructed natural discharge at the Yichang and Datong stations in the dry season in 2006 showed no obvious alterations, and moreover, the reconstructed natural discharge also had a distinct phenomenon of PDDS. Based on the sizes of the red area and the blue area in the dry season, the contributions of dry flow recharge at the Yichang and Datong stations in 2006 were only 18.8% and 6.1%, respectively, and thus, the values of climate variability were 81.2% and 93.9%, respectively. In Fig. 4c,d, although the reconstructed natural monthly average discharge at the Yichang and Datong stations in the dry season in 2011experienced a significant decline in comparison with the figure before restoration, the reconstructed natural discharge also showed a substantial increase compared with that of 1978. Combined with the size of the red area and the blue area in the dry season, the contributions of dry flow recharge in 2011 were up to 40.1% and 27.1% at the Yichang and Datong stations, respectively, and the figures of climate variability were 59.9% and 72.9%, respectively. Clearly, climate variability, rather than dry flow recharge, was still the dominant factor of PDDS.

### During the 156-m and 175-m impoundments

**Figure 5:**
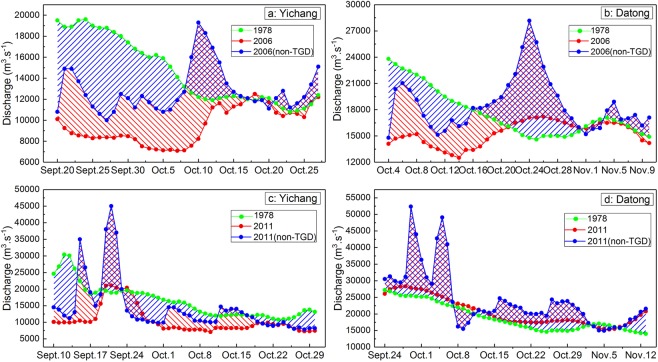
The reconstructed natural monthly average discharge and the actual monthly average discharge at Yichang and Datong stations during the periods of the 156-m impoundment in 2006 and the 175-m impoundment in 2011.

During the periods from September 20 to October 27 in 2006 and from September 10 to October 30 in 2011, the TGD developed the 156-m impoundment and the 175-m impoundment, respectively. As a result, the stored water volumes increased to 108 × 10^8^ m^3^ and 161 × 10^8^ m^3^ in these two periods, respectively, which significantly changed the variation characteristics of the discharge downstream of the TGD. In Fig. 5a–d, the red area shows that the variation in discharge was caused by the 156-m impoundment in 2006 or the 175-m impoundment in 2011, and the blue area means that the variation in discharge was induced by climate variability. Based on the sizes of the red area and the blue area, the contributions of the 156-m impoundment and climate variability were 47.7% and 52.3%, respectively, to the variation in the discharge at Yichang station. At Datong station, the contributions were 37.7% and 62.3%, respectively. Similarly, the daily discharge at the Yichang and Datong stations (Fig. 5d) in 2011 without the TGD were also reconstructed by the Mike 11-HD model. Thus, the 175-m impoundment and climate variability accounted for 44.2% and 55.8% of the discharge changes at Yichang station, respectively. For Datong station, the contributions were 45.4% and 54.6%, respectively. Clearly, climate variability was also the main reason for the variation in discharge downstream of the TGD during the periods of the 156-m and 175-m impoundments.

## Discussion

### Reasons for NDFS

**Figure 6:**
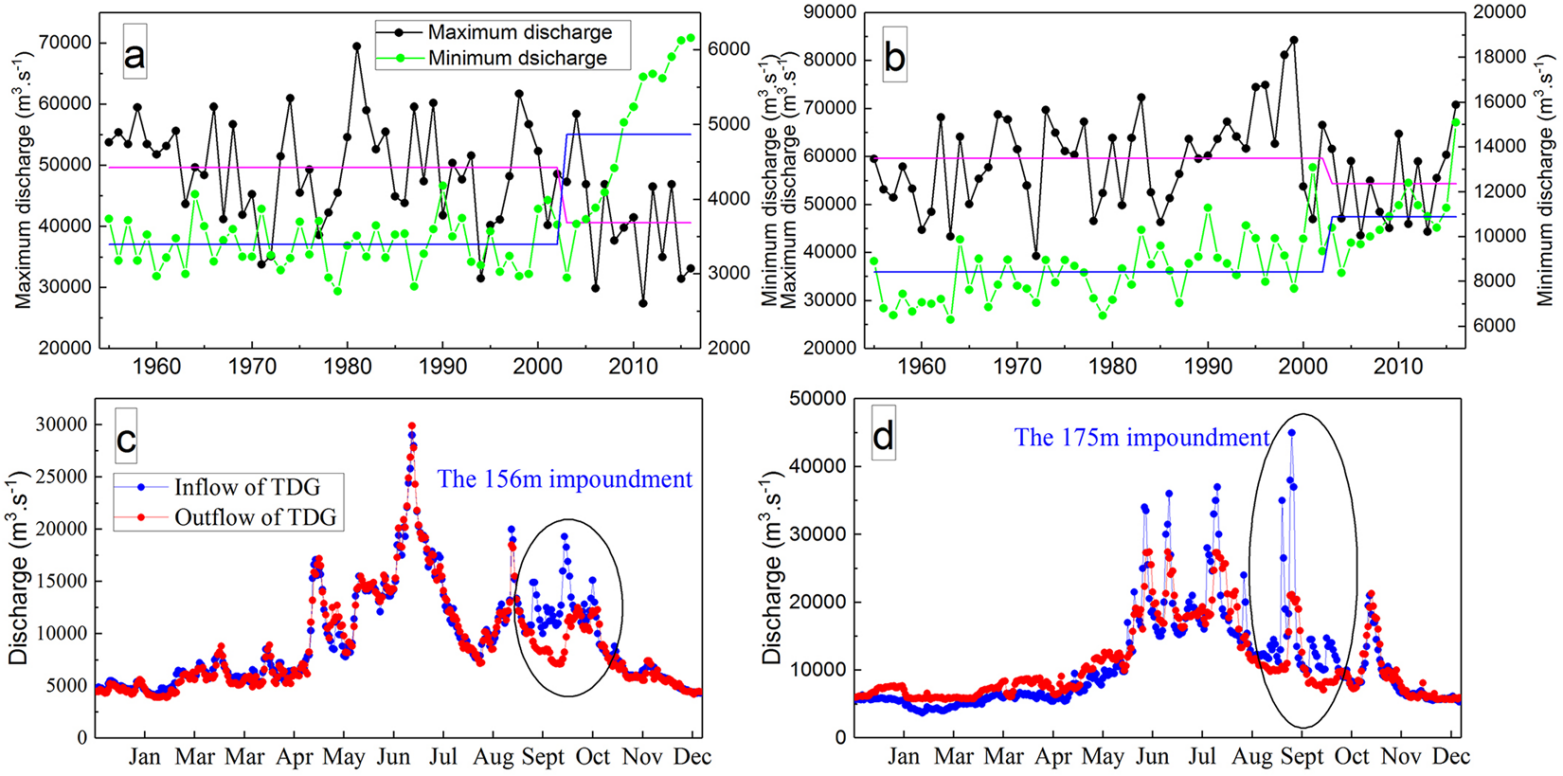
Maximum and minimum discharges at Yichang station (**a**) and Datong station (**b**); inflow and outflow of the TGD in 2006 (**c**) and 2011 (**d**). Note: in (**a**) and (**b**), the pink lines represent the average maximum discharge before and after the operation of the TGD, and the blue lines represent the average minimum discharge before and after the operation of the TGD; (**c**) and (**d**) are the 156-m and 175-m impoundments that occurred at the end of the flood seasons in 2006 and 2011, respectively.

Under the condition of the operation of the TGD, the upstream flood peak will be reduced to prevent downstream floods in the flood season^32–34^, and because of this, the maximum discharge during the post-dam period (2003–2016) shows a significant downward trend in comparison with that during the pre-dam period (Fig. 6a,b). As shown in Fig. 6c,d, the outflow of the TGD was obviously lower than the inflow in 2006 and 2011, which means that the operation of the TGD decreased the discharge in the MLRYR. Furthermore, at the end of the flood season, the TGD implemented the 156-m impoundment and the 175-m impoundment in 2006 and 2011, respectively, which further reduced the discharge downstream from the dam (Fig. 6c,d). Thus, the operation of the TGD was an important reason for the special phenomenon of NDFS in the MLRYR in 2006 and 2011.

**Figure 7:**
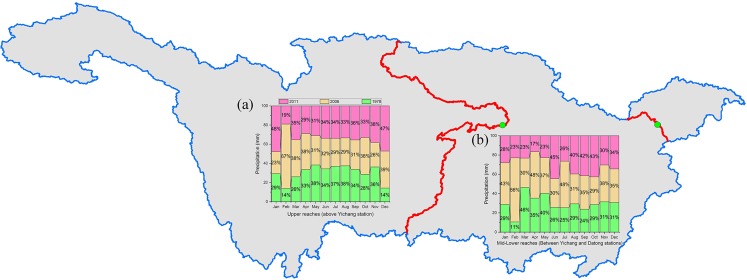
The 100% stacked columns of monthly precipitation in 1978, 2006 and 2011. Note: (**a**) is the precipitation in the upper reaches of the Yangtze River Basin (above Yichang station); (**b**) is the precipitation in the MLRYR (between Yichang and Datong stations).

Figure 7a shows the 100% stacked column of the monthly precipitation in the upper reaches of the Yangtze River Basin (above Yichang station) in 1978, 2006 and 2011. It is clear that the percentage values of monthly precipitation in the flood seasons in 2006 and 2011 decreased significantly in comparison with the values in 1978. As shown in Table 2, the average precipitation during the flood season in the upper reaches in 1978, 2006 and 2011 were 107 mm, 94 mm and 101 mm, respectively. Thus, the precipitation decreased in 2006 and 2011 compared with that in1978, and the reduced precipitation in the upper reaches of the river basin decreased the inflow of discharge to the TGD. Based on the measured discharge data, during the flood season, the average inflow of discharge to the TGD was 12,945 m^3^∙s^−1^ and 14,916 m^3^∙s^−1^ in 2006 and 2001,respectively, and these values were significantly lower than the value in 1978 (19,510 m^3^∙s^−1^); furthermore, combined with the influence of flood peak reduction, the average outflow discharges (Yichang station) further decreased to 12,387 m^3^∙s^−1^ and 13,991 m^3^∙s^−1^, respectively, and thus, the decreasing values were as high as 7123 m^3^∙s^−1^ and 5519 m^3^∙s^−1,^ respectively, compared with the value in 1978 (Table 1). Therefore, the significant decreases of the inflow discharge to the TGD caused by the reduced precipitation and the flood peak reduction caused by the operation of the TGD were the factors that caused NDFS at Yichang station. In Fig. 7b, the percentage values of monthly precipitation in the MLRYR in 2006 and 2011 increased obviously compared with the values in 1978. Table 2 shows the average precipitation during the flood season in the mid-lower reaches in 1978, 2006 and 2011 were 108 mm, 128 mm and 131 mm, respectively. Clearly, the precipitation in 2006 and 2011 increased in comparison with that in 1978, which increased the confluence of tributaries and lakes. Based on the observed discharge data, although the confluence of tributaries and lakes in the MLRYR in 2006 and 2011 increased by 4669 m^3^∙s^−1^ and 2108 m^3^∙s^−1^, respectively, compared with that in 1978, the decreasing values of the average outflow discharge were so large that the average discharge also saw an obvious decrease at Datong station, which led to NDFS at Datong station.

**Table 2:** Monthly precipitation (mm) in the upper and mid-lower reaches of the Yangtze River Basin in 1978, 2006 and 2011.

Month	Upper reaches (above Yichang station)			Mid-lower reaches (between Yichang and Datong stations)		
	1978	2006	2011	1978	2006	2011
Jan.	6.54	5.08	10.66	29.77	44.65	29.02
Feb.	4.87	23.56	6.69	16.81	104.95	36.25
Mar.	16.23	23.98	22.07	108.06	70.59	54.21
Apr.	36.78	41.35	31.70	115.00	157.22	54.04
May.	110.53	88.33	89.43	193.64	177.56	109.18
Jun.	130.18	120.69	128.28	155.48	178.79	268.39
Jul.	145.81	115.20	133.54	93.23	176.87	97.07
Aug.	94.96	73.59	84.64	88.45	92.76	119.32
Sep.	111.58	100.84	117.34	58.01	84.73	101.35
Oct.	48.69	65.73	56.47	58.92	59.35	88.25
Nov.	32.29	22.76	34.08	61.95	75.05	59.76
Dec.	2.77	7.51	9.19	15.60	17.73	17.51

### Reasons for PDDS

Due to the operation of the TGD, the discharge will be supplemented to dramatically relieve downstream drought conditions in the dry season, and thus, the minimum discharge during the post-dam period shows an obvious increasing trend in comparison with the discharge in the pre-dam period (Fig. 6a,b). In Fig. 6c,d, the outflow of the TGD during the dry seasons in 2006 and 2011 was larger than the inflow, which shows that the operation of the TGD increased the discharge downstream of the dam. Therefore, the operation of the TGD was an essential reason for the special phenomenon of PDDS.

As shown in Fig. 7a, the percentage values of monthly precipitation during the dry seasons in 2006 and 2011 increased significantly compared with the values in 1978. Thus, the increased precipitation increased the inflow of discharge to the TGD. According to the measured data, the average inflow of discharge to the TGD in the dry season in 2006 was as high as 5870 m^3^∙s^−1^, and the inflow and outflow of the TGD in the dry season in 2006 were almost the same (Fig. 6c), which led to the average outflow discharge showing no significant alteration, i.e., approximately 506 m^3^∙s^−1^ higher than the value in 1978. Hence, the main reason for PDDS at Yichang station in 2006 was the rich inflow to the TGD caused by the increased precipitation, and the dry flow recharge had a small effect. In contrast, the average inflow discharge in 2011 was as high as 6397 m^3^∙s^−1^ due to the rich precipitation, and the outflow of the TGD was apparently larger than the inflow in the dry season (Fig. 6d). Finally, the average outflow discharge was further increased to 7475 m^3^∙s^−1^, with approximately 2370 m^3^∙s^−1^ more than the value in 1978(Table 1). Overall, the rich inflow to the TGD caused by the increased precipitation and the dry flow recharge caused by the operation of the TGD both resulted in PDDS at Yichang station in 2011.

In Fig. 7b, the percentage values in the MLRYR in 2006 and 2011 showed a significant increasing trend compared with the values in 1978, which increased the confluence of tributaries and lakes. Based on the observed discharge data, the supplementation of tributaries and lakes in the MLRYR in 2006 and 2011 increased by 2741 m^3^∙s^−1^ and 519 m^3^∙s^−1^, respectively, combined with the increased average discharge at Yichang station (506 m^3^∙s^−1^ and 2370 m^3^∙s^−1^, respectively), both of which led to an obvious increase in the average discharge during the dry season at Datong station in 2006 and 2011. In conclusion, the rich inflow to the TGD and the rich confluence of tributaries and lakes, which were both caused by the increased precipitation, were the reasons for PDDS at Datong station in 2006 and 2011, and the influence of dry flow recharge caused by the operation of the TGD operation had a small effect on this phenomenon in 2006 but was an important factor in 2011.

### Is the operation of the TGD be responsible for the extreme drought event?

The influence of the operation of the TGD on the extreme drought events downstream of the dam was mainly reflected in two aspects, namely, indirect and direct influences. Indirect influences reflect meteorological drought: The operation of the TGD changed the spatial and temporal distribution of runoff and thus influenced climate variation in the Yangtze River Basin. Climate variation can influence runoff processes. Based on the existing research results, the operation of the TGD had little influence on climate change. For instance, from June to August 2006, the TGD increased the average temperature and precipitation by only approximately 0.01 °C and 1.7% in the TGD area^34^. Apparently, the indirect influence of the TGD on the variation in discharge was small. Direct influences reflect hydrological drought: Under the effects of the operation of the TGD, the outflow was completely different from the inflow, which directly changed the discharge process in the MLRYR. These effects are much more swift and dramatic. Therefore, we analysed only the direct influence on the extreme drought events in 2006 and 2011.

An extreme drought event occurred in the Yangtze River basin from June to August 2006, which caused severe economic losses. In these three months, the average daily inflow and outflow discharge was nearly equal (Fig. 6c); thus, the influence of the operation of the TGD on the variation in discharge in the MLRYR was small. Thus, the operation of the TGD in these months did not significantly aggravate the drought condition and was not responsible for the extreme drought event in 2006. From March to May 2011, the average precipitation in the MLRYR decreased to the lowest point in history due to the extreme drought event, and it was equal to approximately 53.1% of the values in the normal years. As shown in Fig. 6d, the average daily outflow discharge was obviously higher than the average daily inflow discharge from March to May, and thus, the total water volume supplemented by the TGD was as high as 36.7 × 10^8^ m^3^ in this period. Apparently, the operation of the TGD significantly alleviated the extreme drought conditions and thus was not be responsible for the extreme drought event in the MLRYR in 2011.

## Conclusions

Analysing the variation characteristics of streamflow and estimating the effects of climatic variation and human activities are meaningful for future water resource planning and management decisions, especially when extreme hydrological events occur. During extreme drought years, the discharge process in the MLRYR (in the post-dam period) was obviously changed due to the effects of both the operation of the TGD and the extreme drought compared with the process in the pre-dam period. The measured data and the Mike-11HD model were both used to analyse the variation characteristics of runoff and its causes. The main conclusions are as follows:

1. In the dry season, the discharge in 2006 and 2011 increased significantly compared with that in 1978 and 1986. In the flood season, the average discharge at the Yichang, Zhicheng, Shashi and Hankou stations in 2006 and at the Luoshan and Datong stations in 2011 all decreased to the lowest point in history. Distinctly, the unusual phenomenon of “NDFS-PDDS” occurred in 2006 and 2011.
2. The decreased inflow of discharge to the TGD caused by climate variability and the flood peak reduction caused by the TGD both caused the phenomenon of “NDFS”. The rich inflow of the TGD and the rich confluence of tributaries and lakes both induced by climate variability were the reasons for the phenomenon of “PDDS”, and the influence of dry flow recharge caused by the TGD was small for this phenomenon in 2006 but was an important factor in 2011.
3. Whether there is an indirect or direct influence of the operation of the TGD, the TGD was not responsible for the extreme drought events in the MLRYR in 2006 and 2011. In particular, from March to May 2011, the TGD operation obviously alleviated the drought.
4. Based on the Mike 11-HD model, the contributions of the operation of the TGD and climate variability on variations in discharge in 2006 and 2011 were calculated compared with the discharge in 1978. The results show that climate variability was the dominant factor for the discharge changes downstream of the TGD. During the periods of the 156-m impoundment in 2006 and the 175-m impoundment in 2011, surprisingly, climate variability was still the dominant factor causing the variation in discharge downstream of the reservoir.

With the joint operation of reservoirs in the Yangtze River Basin, the phenomenon of “NDFS-PDDS” will be more frequent during extreme drought years and should receive more attention. In the next step, we will concentrate on the effects of the phenomenon on natural ecosystems to improve basic scientific understanding and mindful management of river systems at multiple scales. The insufficiency of this study is that we did not take other human activities (e.g., human water consumption and water-soil conservation projects) into consideration, which might overestimate the effects of climate change, and thus, the effects of other human activities need further study.

## Methods

To estimate the effects of the operation of the TGD and climate variability on the variation in discharge, we used the Mike 11-HD model (for more details, see SI) to reconstruct the natural discharge by assuming no flow regulation from the TGD during the post-dam period. After that, the contributions of the operation of the TGD and climate variability on discharge change can be calculated by eqs (1) and (2).

1 $${R}_{TGD}=\frac{|{Q}_{r(a)}-{Q}_{m(a)}|}{|{Q}_{r(a)}-{Q}_{m(a)}|+|{Q}_{r(a)}-{Q}_{m(b)}|}$$

2 $${R}_{Climate}=\frac{|{Q}_{r(a)}-{Q}_{m(b)}|}{|{Q}_{r(a)}-{Q}_{m(a)}|+|{Q}_{r(a)}-{Q}_{m(b)}|}$$

where ${R}_{TGD}$ and ${R}_{Climate}$ denote the contributions of the TGD operation and climate variability on the variation in discharge, respectively; ${Q}_{r}$ represents the reconstructed natural discharge; ${Q}_{m}\,$represents the actual discharge; the subscript “a” is the extreme drought year after the operation of the TGD, namely, 2006 or 2011; and the subscript “b” is the extreme drought year before the operation of the TGD, namely, 1978. Note: the shortcoming is that apart from the effects of the operation of the TGD and climate variability, there are other potential influencing factors, such as human water consumption and water and soil conservation, that are not taken into consideration in this study due to the lack of data.

### Data collection

In this study, data on daily discharge and water levels from 1955–2016 at the main hydrological stations in the mid-lower reaches of the Yangtze River (MLRYR) were provided by the CWRC (Ministry of Water Conservancy of China). The daily inflow of discharge to the TGD in 2006 and 2011was obtained from the China Three Gorges Corporation (http://www.ctg.hk/sxjt/sqqk/index.html). The river topographic data between Yichang and Datong in 2011were received from the Hydrological Bureau of Changjiang Water Conservancy Commission. The daily precipitation at 145 weather stations in 1978, 2006 and 2011 was provided by the Resource and Environment Data Cloud Platform. To analyse the variation characteristics of discharge in 2006 and 2011 under the influence of both extreme drought and TGD, the hydrological data of 2006 and 2011 (i.e., post-dam period) were compared with the extreme drought years of 1978 and 1986 (i.e., pre-dam period).

## Supplementary information

Supplementary
