# Supplementary material for: Influence of Climate Variability and Reservoir Operation on Streamflow in the Yangtze River
Source: Sci Rep. 2019 Mar 25;9:5060. doi: 10.1038/s41598-019-41583-6 (PMC6433911; doi:10.1038/s41598-019-41583-6)
Supplement: Supplementary file 1 — Supplementary [file 41598_2019_41583_MOESM1_ESM.pdf]

Influence of Climate Variability and Reservoir Operation on Streamflow in the Yangtze River

Yuanfang Chai[1], Yitian Li[1], Yunping Yang[2,✉], Boyuan Zhu[3], Sixuan Li[1], Can Xu [1], Congcong Liu[1]

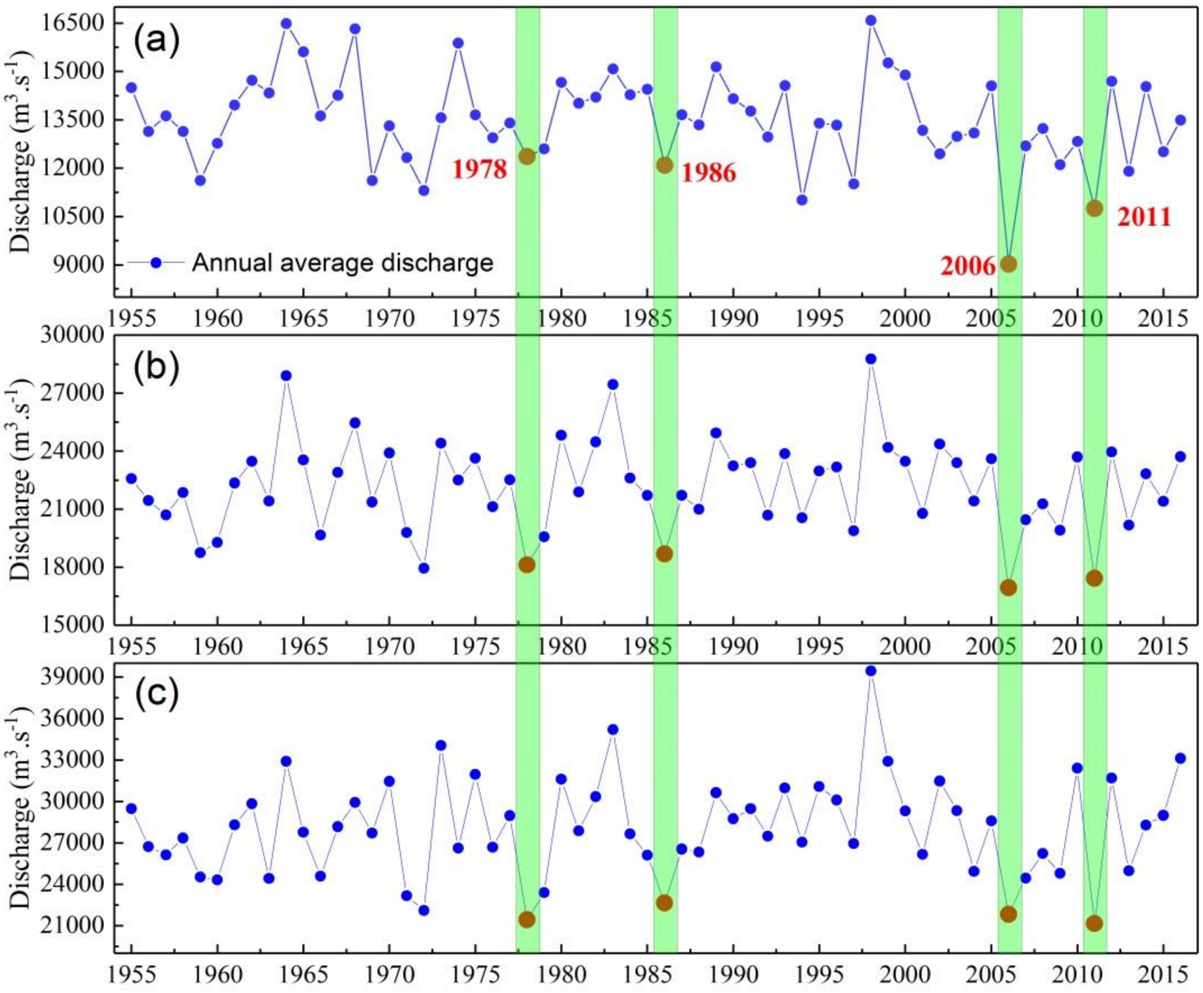

Supplementary Figure S1.The annual average discharge at the main hydrologic stations of the Yangtze River; Note: (a) Yichang station; (b) Hankou station; (c) Datong station; Datong station is the last hydrologic station in the Yangtze River Basin, and thus its runoff can represent the total water resources of the river basin.

Mike 11-HD model

Governing equation

The Mike 11-HD model was developed by the Danish Hydraulic Institute (DHI) and is used for calculating unsteady flow, discharge and water levels in rivers and channels[1]. The governing equations of this model are the Saint-Venant equations (Equations (1) and (2)), which are dispersed by the finite difference method. The difference scheme is the six points, central, implicit, andfinite-difference numerical scheme (Abbott-Ionescu) and the numerical method adoptsthechasing method.

Continuity equation:

$$\frac{\partial A}{\partial t}+\frac{\partial Q}{\partial x}=q \qquad (1)$$

Momentum equation:

$$\frac{\partial \mathrm{Q}}{\partial \mathrm{t}}+\frac{\partial}{\partial \mathrm{x}}\left(\propto \frac{\mathrm{Q}^2}{\mathrm{A}}\right)+\mathrm{g.A.}\frac{\partial \mathrm{h}}{\partial \mathrm{x}}+\frac{n^2\mathrm{gQ|Q|}}{\mathrm{A.R}^{4/3}}=0 \qquad (2)$$

where A (m^2) is the cross-sectional area; t (s) is the time; Q ($m^2 \cdot s^{-11}$) represents the discharge; x (m) is the downstream direction; q ($m^2 \cdot s^{-1}$) means the lateral inflow; $\propto$ (/) is the momentum distribution coefficient; g ($m^2 \cdot s^{-1}$) denotes the acceleration due to gravity; h (m) is the water level above a reference datum; n ($s \cdot m^{-1/3}$) is the Manning resistance coefficient; and R (m) is the hydraulic radius.

Boundary conditions and model validation

Boundary conditions include the inflow boundary condition, the lateral inflow and outflow boundary conditions and the outflow boundary condition. The reach in the model is the stretch from the Yichang station to the Datong station, where the topographic data are from 2011. Before reconstructing the natural discharge (non-operation of the TGD) in 2006 and 2011, we first needed to calibrate and validate the model; taking the extreme drought year of 2011 as an example, the inflow boundary condition adopted the daily discharge at Yichang station in 2011. The lateral outflow boundary condition used the daily discharges at Songzikou, Taipingkou and Ouchikou in 2011 (the runoff of the Yangtze River flows into Dongting Lake by these three mouths). The lateral inflow boundary condition adopts the daily discharges at the Chenglingji, Huangzhuang and Hukou hydrologic stations in 2011 (the runoff of Dongting Lake, Hanjiang River and Poyang Lake flows into the Yangtze River by these three hydrologic stations, respectively). The outflow boundary condition used the water level-discharge curve (Q-H) at Datong in 2011. Based on the above boundary conditions, the model was calibrated and validated by comparing the actual water level and discharge (Fig. S1). As shown in Fig. S1, it is quite clear that the simulated water and discharge are in good agreement with the actual data, which means that the model has high reliability.

Under the condition of non-operation of the TGD, the daily inflow of discharge to the TGD will all flow into the MLR. Thus, after calibration and validation, the inflow boundary condition, namely, the daily discharge at Yichang station in 2011 (still taking the year of 2011 as an example), was replaced by the daily inflow of discharge to the TGD in 2011, and the other boundary conditions remained the same. By doing so, the natural discharge in the MLR in 2011 can be constructed. Based on the model, the natural discharge in the MLR in 2006 was also reconstructed.

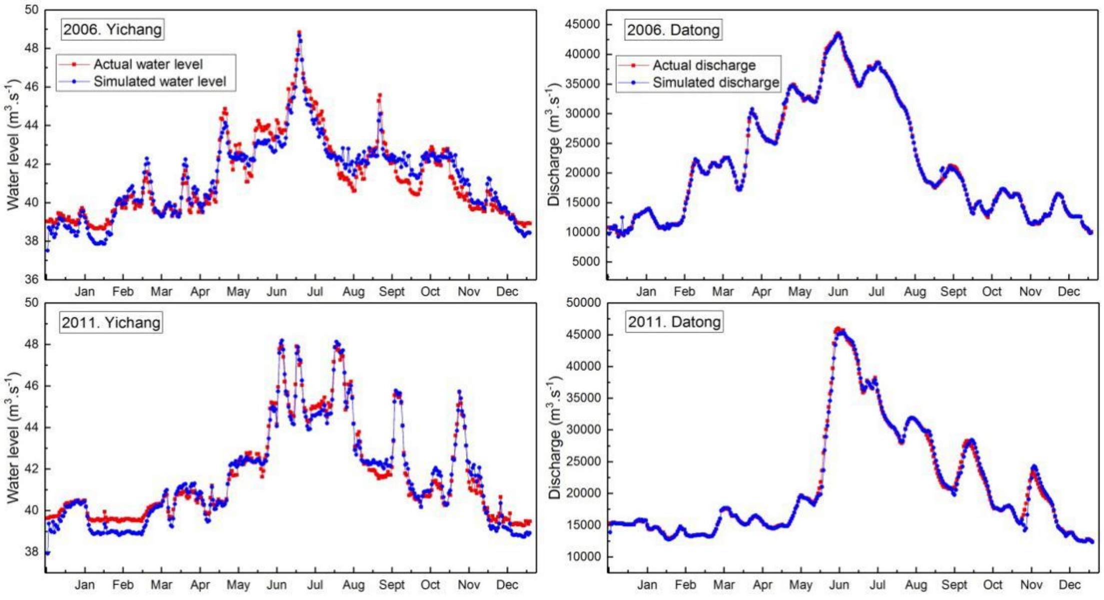

Supplementary Figure S2. The actual water level (Yichang) and discharge (Datong) and the simulated water level and discharge in 2006 and 2011.

Reference

1. Doulgeris, C., Georgiou, P., Papadimos, D. & Papamichail, D. Ecosystem approach to water resources management using the MIKE 11 modeling system in the Strymonas River and Lake Kerkini . *J Environ Manage*. **94(1),** 132-143 (2012).
